# Rationalising blood tests in a resource-limited emergency unit: A quality improvement project

**DOI:** 10.4102/safp.v67i1.6067

**Published:** 2025-02-06

**Authors:** Renette Esterhuizen, Lilani I. Tribelhorn, Danielle M.J. Thomas, Eugenne Elliott, Talat Habib, Arun Nair

**Affiliations:** 1 Northern Cape Department of Health, Faculty of Family Medicine, Robert Mangaliso Sobukwe Hospital, Kimberley, South Africa; 2 National Health Laboratory Service, Robert Mangaliso Sobukwe Hospital, Kimberley, South Africa; 3 Department of Family Medicine, Robert Mangaliso Sobukwe Hospital, Kimberley, South Africa; 4 Department of Family Medicine, Faculty of Health Sciences, University of the Free State, Bloemfontein, South Africa

**Keywords:** blood tests, emergency unit, quality improvement, resource utilisation, evidence-based guidelines

## Abstract

**Background:**

In resource-limited emergency settings, blood testing is crucial for diagnostics but can lead to financial strain and diagnostic challenges if used indiscriminately. This quality improvement project (QIP) aimed to identify commonly requested blood tests in an emergency department (ED), assess their appropriateness, and establish evidence-based guidelines for judicious use. This project was conducted during the family medicine rotation of interns at a tertiary hospital in a semi-urban area.

**Methods:**

The Plan-Do-Study-Act (PDSA) method was employed. Pre- and post-intervention data were collected. An educational intervention, featuring informative visual aids was implemented to help guide the medical practitioners in the unit on appropriate blood test ordering.

**Results:**

The intervention led to a significant 48.7% reduction in total daily blood tests ordered, with a notable decrease in full-panel requests and an increase in individual test ordering.

**Conclusion:**

Educational interventions, aimed at guiding blood test requests, can significantly reduce unnecessary testing. Long-term data collection is necessary to confirm sustained changes in practice.

**Contribution:**

Our findings indicate that clear, evidence-based guidelines for the judicious use of blood tests can positively impact test ordering, particularly in resource-limited settings, and suggest opportunities for further long-term studies.

## Introduction

### Background

The healthcare system in all countries face resource constraints and financial limitations, making judicious use of medical resources essential. In emergency settings, laboratory testing plays a vital role in diagnosis and management, but overutilisation of blood tests can strain resources and negatively impact patient care.

Over-utilisation of laboratory tests is a global issue.^1^ Studies indicate that many tests are ordered unnecessarily, contributing to increased healthcare costs without improving patient outcomes.^1,2,3^ Factors such as diagnostic uncertainty, patient expectations, fear of litigation and a lack of awareness about test costs drive over-utilisation.^3^

Full blood counts (FBCs), U&E (urea, creatinine and electrolyte) panels and other tests are frequently over-ordered, often without significantly impacting patient management.^3^ The annual report from the National Health Laboratory Service (NHLS) shows that tests such as FBC, U&E and CRP (C-reactive protein) all featured in the top six most ordered blood tests in South Africa during 2022–2023.^4^

Educational initiatives and administrative changes have been shown to reduce inappropriate test ordering.^5,6,7^ Programmes such as ‘Choosing Wisely’ advocate for the judicious use of medical resources.^8^ There is a lack of guidelines to aid the medical practitioner in South Africa on how to order bloods sensibly.^9^ This Quality Improvement Project (QIP) conducted by interns during their family medicine rotation sought to guide practice by developing and implementing evidence-based guidelines to optimise blood test utilisation in a resource-limited emergency unit. A literature review was compiled to identify common blood tests ordered in similar healthcare settings and strategies to reduce over-utilisation were identified.

## Method

The project was conducted at the Emergency Centre Gateway Unit 2 of Robert Mangaliso Sobukwe Hospital, Kimberley, Northern Cape, South Africa. It is a tertiary medical facility in a semi-urban area. Unit 2 handles an average of 3500 patients monthly and includes multiple consultation areas and a short-stay area for temporary admissions. Green and yellow triage patients are assessed here before being discharged, referred to specialised departments or admitted for further care.

In this project, we used interventional and observational methodologies by employing the Plan-Do-Study-Act (PDSA) method^10^ to test the impact of our intervention to improve practices in the current clinical setting.

This QIP study was performed during the intern’s family medicine rotation in their second year of internship between 03 July 2023 and 30 September 2023. Data were collected over two distinct periods: a 2-week pre-intervention phase and a 1-week post-intervention phase. As a result of significant time constraints in a pressing internship schedule with frequent departmental rotations, the duration of the second data collection period was shortened.

Pre-intervention data collection involved recording all blood tests requested over a 2-week period. Medical practitioners were required to manually log their own barcodes in a paper-based logbook by pasting the relevant barcodes in a book with tables that included fields for the patient’s sticker, diagnosis and barcode. To ensure completeness, we cross-referenced the unit’s statistical Excel sheet, which tracks patient data, to identify any missing barcodes. Blood tests conducted on patients but not logged were added retrospectively using the patient’s hospital number and the NHLS’ TrakCare database of blood results. Each blood test was then categorised, grouped and aggregated to determine the total count.

**FIGURE 1:**
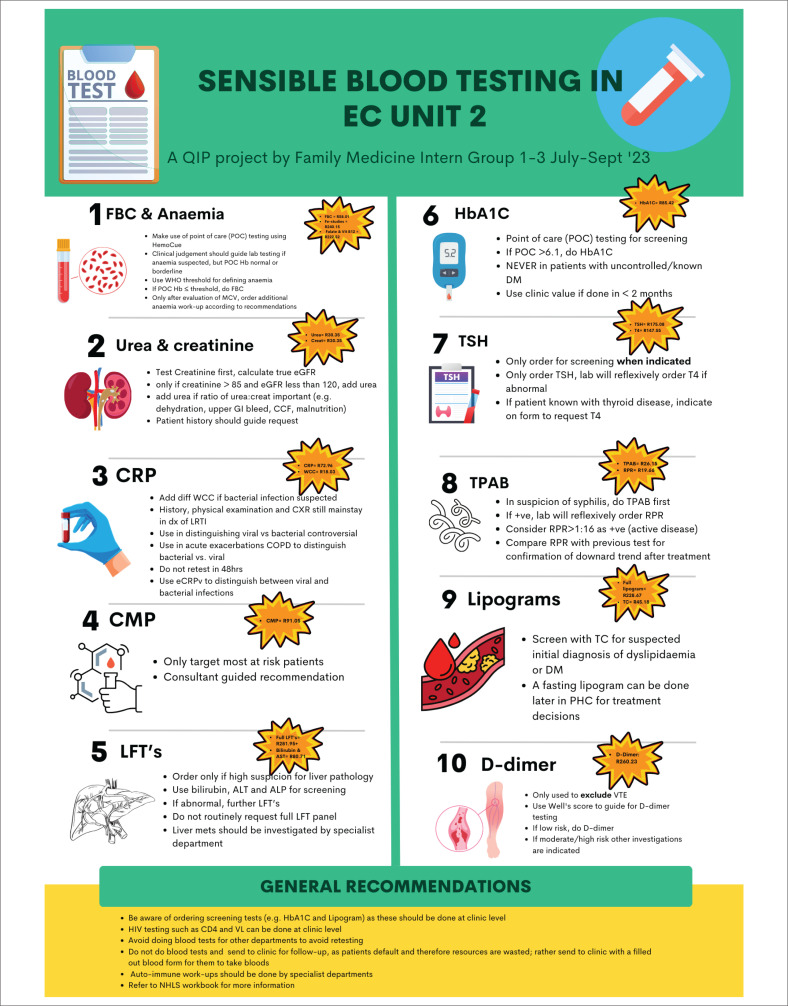
Visual aid poster for rational blood testing in the emergency unit.

An educational session complemented by the display of visual aids (posters) was conducted to disseminate guidelines on appropriate blood test ordering to all medical practitioners working in the unit during the study period, including interns, medical officers and consultants. The guideline, primarily developed by the corresponding author, with input from the other authors, was based on a literature review compiled before the study. It drew on numerous resources from studies in similar settings and units. A condensed version of the guideline was compiled into a visually engaging poster (Figure 1) and displayed prominently throughout the unit to facilitate ease of reference. The guideline advised against selecting entire panels of tests unless necessary and recommended requesting individual tests on the forms instead. It also emphasised the importance of evaluating if the test would influence acute patient management for the current visit.

Data were collected in the same way as before, for a 1-week post-intervention period to measure the impact of the educational intervention on the unit’s ordering of blood tests.

### Ethical considerations

Given that this project was conducted as a quality improvement project (QIP) within the framework of an internship programme and was part of the requirements for successfully completing the internship, there was implied consent by the institution and the Department of Family Medicine to carry out this project. Therefore, higher ethical research clearance was deemed unnecessary. However, the project adhered to institutional guidelines and ethical standards throughout its implementation.

## Results

The intervention resulted in a 48.7% decrease in total daily blood tests ordered. When looking at the three major contributors to pre-intervention blood tests – FBC, U&E and CRP – the researchers observed a significant reduction following the intervention.

The data showed a shift in blood test ordering practices, with a decrease in full-panel requests for FBCs and an increase in requests for individual components such as haemoglobin, differential, white cell count and haematocrit. Before the intervention, 87% of all FBC requests were for full panels, compared to 79% post-implementation, indicating more targeted test ordering. However, this trend was not observed with U&Es, where the proportion of full-panel requests increased from 96% to 100% of all U&E requests post-implementation. This indicates that fewer individual components of the U&E were being requested after the intervention. Nevertheless, the total number of full U&E panels requested per day decreased by 52.5% post-implementation, demonstrating more conservative requesting by medical practitioners.

**FIGURE 2:**
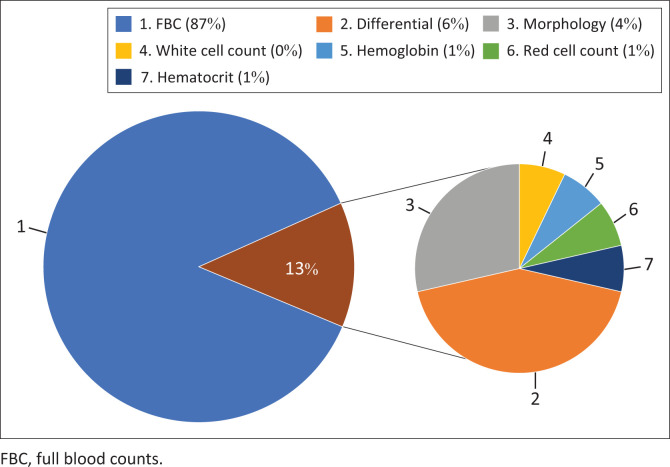
Distribution of full-panel versus individual component requests for full blood counts in pre-intervention testing.

**FIGURE 3:**
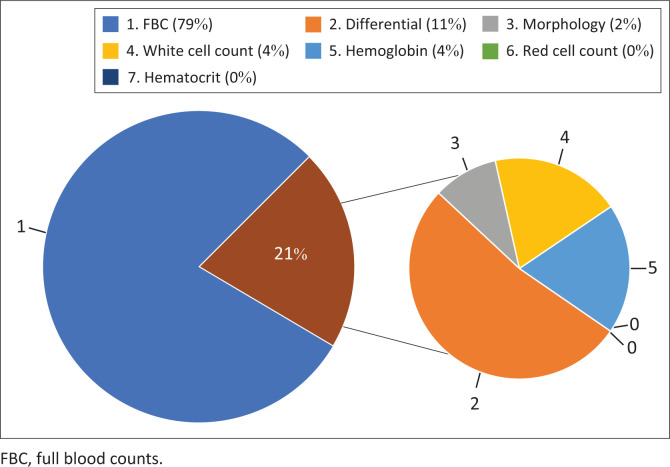
Distribution of full-panel versus individual component requests for full blood counts in post-intervention testing.

The difference in the pre- and post-implementation ordering of FBC and components can be seen in Figure 2 and Figure 3.

Regarding CRP tests, the percentage requested relative to the total samples sent decreased from approximately 5.74% to 1.77% in the post-implementation phase. This indicated that medical practitioners were employing a more evidence-based rationale when ordering tests that do not alter patient management in an acute setting.

## Discussion

The findings suggest that targeted educational interventions can effectively reduce unnecessary blood tests in resource-limited settings, potentially freeing resources for other utilisation and patient care. Through academic discussions supplemented with visual aids and context-specific guidelines, we demonstrated that medical practitioners could change their habits regarding the ordering of routine blood tests.

However, the short-term nature of this study is a potential limitation, and follow-up studies are necessary to confirm sustainability of these practices. To address the difference in duration of the pre- and post-intervention data collection periods, we standardised our totals on a per-day basis.

We acknowledge the limitations of having junior doctors develop a poster to guide blood testing practices, as it may lack the depth and standardisation required for widespread applicability. However, our observations highlight a clear need for practical guidance in this area, particularly in resource-limited emergency settings where over- or under-utilisation of blood tests can have significant consequences. This underscores the importance of developing formal, evidence-based guidelines tailored to the unique challenges and priorities of South African emergency centres. Such guidelines would ensure consistency, improve diagnostic efficiency and optimise resource utilisation in these critical settings.

We were unable to determine whether the shift towards judicious blood test requesting had any adverse impact on patient outcomes. This limitation arises from the retrospective nature of the study and the absence of specific data linking test utilisation to clinical harm. Further research, including prospective studies, would be required to assess the safety and clinical implications of these interventions.

Another limitation is the potential for self-report bias, as medical practitioners were responsible for logging their own barcodes. We attempted to mitigate this by using the unit’s patient statistic log keeping tool, an Excel-based tracking tool where every patient entering the unit is recorded, including name, hospital number and diagnosis. This tool ensures that all patients seen in the unit are accounted for, allowing us to cross-check and identify any blood requests that were not manually logged.

Future research should focus on long-term data collection and methods to reduce biases. Developing comprehensive guidelines tailored to economically restricted settings by trusted organisations could further enhance the standard of care in South African emergency centres.

## Conclusion

This QIP highlights the impact of educational interventions in rationalising blood test utilisation in a resource-limited emergency unit. The significant reduction in unnecessary blood tests underscores the potential for such interventions to improve resource utilisation and patient outcomes. Long-term data and further studies are essential to confirm the sustained efficacy of these changes.
